# *Moringa oleifera* Lam Leaf Extract Stimulates NRF2 and Attenuates ARV-Induced Toxicity in Human Liver Cells (HepG2)

**DOI:** 10.3390/plants12071541

**Published:** 2023-04-03

**Authors:** Siqiniseko S. Ndlovu, Anil A. Chuturgoon, Terisha Ghazi

**Affiliations:** Discipline of Medical Biochemistry, School of Laboratory Medicine and Medical Sciences, University of KwaZulu-Natal, Durban 4041, South Africa

**Keywords:** *Moringa oleifera*, antioxidants, antiretroviral drugs, oxidative stress, mitochondrial toxicity, NRF2 signalling

## Abstract

The World Health Organization (WHO) reported that there are 37 million individuals living with the human immunodeficiency virus (HIV) worldwide, with the majority in South Africa. This chronic disease is managed by the effective use of antiretroviral (ARV) drugs. However, with prolonged use, ARV drug-induced toxicity remains a clinically complex problem. This study investigated the toxicity of ARV drugs on mitochondria and the NRF2 antioxidant pathway and its possible amelioration using *Moringa oleifera* Lam (MO) leaf extracts. This medicinal plant has a range of functional bioactive compounds. Liver (HepG2) cells were treated with individual ARV drugs: Tenofovir disoproxil fumarate (TDF), Emtricitabine (FTC), and Lamivudine (3TC) for 96 h, followed by MO leaf extracts for 24 h. Intracellular ROS, cytotoxicity, lipid peroxidation, total and reduced glutathione (GSH), ATP, and mitochondrial polarisation were determined. Finally, protein (pNRF2, NRF2, SOD2, CAT, and Sirt3) and mRNA (*NRF2*, *CAT*, *NQO1 SOD2*, *Sirt3*, and *PGC1α*) expression were measured using Western blot and qPCR, respectively. TDF, FTC, and 3TC significantly increased intracellular ROS and extracellular levels of both MDA and LDH. ARVs also reduced the GSH and ATP levels and altered the mitochondrial polarization. Further, ARVs reduced the expression of *NRF2 SOD2*, *Sirt3*, *CAT*, *NQO1*, *UCP2* and *PGC1α* mRNA and consequently pNRF2, NRF2, SOD2, Sirt3 and CAT protein. In contrast, there was a significant reduction in the extracellular MDA and LDH levels post-MO treatment. MO significantly reduced intracellular ROS while significantly increasing GSH, ATP, and mitochondrial membrane polarization. The addition of MO to ARV-treated cells significantly upregulated the expression of *NRF2*, *SOD2*, *Sirt3*, *CAT*, *UCP2*, *PGC1α*, and *NQO1* mRNA and pNRF2, NRF2, SOD2, Sirt3 proteins. Thus, MO ameliorates ARV-induced hepatotoxicity by scavenging oxidants by inducing the NRF2 antioxidant pathway. MO shows great therapeutic potential and may be considered a potential supplement to ameliorate ARV drug toxicity.

## 1. Introduction

Human immunodeficiency virus (HIV) remains a major public health issue globally, with almost 36 million deaths so far [1]. The World Health Organization (WHO) reports that there are 38 million individuals currently living with HIV worldwide, and most of these individuals are in South Africa (SA) [1,2]. SA is among the top five countries with the highest HIV infection prevalence (19%), having about eight million people living with HIV [2,3] bearing the largest burden [4]. HIV is managed by the effective use of antiretroviral (ARV) drugs. Over the past years, ARV drug formulations have been improved and combined into two or three ARV drug classes to make highly active antiretroviral therapy (HAART), also known as antiretroviral therapy (ART).

The key motive of HAART is enhanced efficiency, in which viral replication is suppressed through the co-administration of different classes of drugs that inhibit replication by several mechanisms and at multiple stages [5,6,7]. HIV-infected people need to be on HAART for life to keep the viral load suppressed. This makes HAART a life-long treatment, and the long-term use of these drugs has led to the emergence of adverse health outcomes, particularly changes to metabolic parameters, lipodystrophy, dyslipidemia, and hepatic steatosis [8,9,10,11,12].

Studies into HAART and their associated disorders show that individual ARV drugs have different degrees of toxicity that are tissue-specific and time-dependent [13,14]. Oxidative stress and mitochondrial dysfunction are highlighted as key metabolic pathways by which ARV drug-induced toxicity arises [15,16,17,18].

Both oxidative stress and mitochondrial impairment result from xenobiotic metabolism and accompany one another [14,19]. Disruptions to mitochondrial function increase the production of reactive oxygen species (ROS) through impaired oxidative phosphorylation [20]. Increased free radical production, over a period of time, depletes the antioxidant defense response, eventually resulting in oxidative damage to biomacromolecules [21,22]. The primary defense against oxidative stress in cells is the induction of the antioxidant GSH, which scavenges ROS and dampens oxidative damage to macromolecules [23,24]. Nuclear factor erythroid 2 p45–related factor-2 (NRF2) is a major transcription factor that stimulates the antioxidant response element (ARE), a regulatory element found in the promoters of several cytoprotective and antioxidant genes, including NAD(P)H:quinone oxidoreductase-1 (NQO1), superoxide dismutase (SOD) and catalase (CAT) [25,26,27]. At homeostasis conditions, NRF2 is kept in the cytoplasm and constantly degraded through Kelch-like ECH-associated protein-1 (Keap1) ubiquitination. Under oxidative stress, elevated ROS oxidizes specific cysteine residues in Keap-1, weakening its affinity towards NRF2. Keap1 then dissociates from the Neh2 domain, enabling the accumulation and phosphorylation of NRF2 in the cytosol. The phosphorylated NRF2 then translocates to the nucleus, where it dimerizes with small Maf proteins and binds to the ARE, promoting the transcription of antioxidant genes [28,29].

As much as ARV formulations have been improved, they are not void of side effects. There is growing evidence that the newer ARVs, as per current South African guidelines: Tenofovir disoproxil fumarate (TDF), Lamivudine (3TC)/Emtricitabine (FTC), and Dolutegravir (DTG) [30] exhibit toxicity. TDF, FTC, and 3TC have been shown to induce oxidative stress and mitochondrial toxicity [31,32,33]. To minimize these toxicities, ARV formulation should be continuously assessed and improved to a safer, optimum therapy without compromising its efficacy. Alternatively, the use of supplements or an adjuvant may be beneficial. *Moringa oleifera* Lam (MO), a medicinal plant that belongs to the Moringaceae family, is found throughout SA and is identified for its nutritious and traditional medicinal uses. All parts of this plant have a notable range of pharmaceutical and nutritional properties.

MO leaf extract is rich in flavonoids such as quercetin, kaempferol, isorhamnetin, and apigenin [34,35]. Polyphenols found in MO are gallic acid, catechin, epicatechin, p-coumaric acid, ferulic acid, vanillin, caffeic acid, protocatechuic acid, cinnamic acid, procyanidins, aurantiamide acetate, quercetin glycoside, quercetin rhamnoglycoside, pterygospermin, and chlorogenic acid. Lastly, MO leaves are rich in minerals such as calcium, potassium, zinc, magnesium, iron, and copper, and vitamins including vitamins A, B, pyridoxine, nicotinic acid, and folic acid, C, D, and E [35,36,37,38,39]. Therefore, MO leaves provide powerful antioxidative benefits [40,41], free radical scavenging [42], anticancer [43,44], hepatoprotective [45], anti-proliferative, anti-mutagenic, promotes carbohydrate metabolism [46], and repairs DNA [43] thus validating the traditional claims.

In this study, the HepG2 cell line was used because they are a suitable and well-characterized model of the human liver since they have similar physiological functions to primary hepatocytes [47]. They have been used in previous studies evaluating the hepatoprotective effects of medicinal plants [48,49,50] and the effect of antiretroviral drugs on mitochondrial toxicity and oxidative stress [21,51,52,53,54]. Most importantly, the liver is the metabolic hub of humans and is abundant in mitochondria [55]. HepG2 cells possess cytochrome P450 activity and have hence been identified as an early model for metabolism-associated drug-induced liver toxicity [56,57,58].

This study investigates the toxicity of the current generation of ARV drugs on the mitochondria and NRF2 antioxidant pathway. TDF, FTC, and 3TC were used because they are nucleotide reverse transcriptase inhibitors and the cornerstone of HAART on the SA’s recommended guideline [59,60]. This study further investigates the potential of aqueous MO leaf extract to ameliorate ARVdrug-induced toxicity in human liver cells through the regulation of the NRF2 antioxidant signaling mechanism.

## 2. Results

### 2.1. Oxidative Stress and Cellular Membrane Integrity

#### 2.1.1. Induction of Oxidative Stress

**Figure 1:**
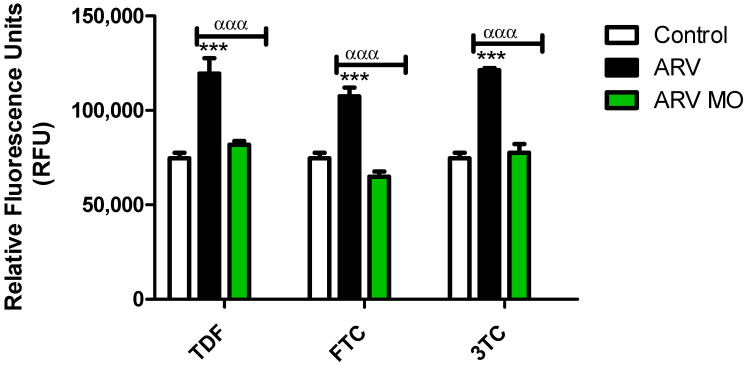
Assessment of ROS generation post-ARV and post-ARV MO treatment. TDF, FTC, and 3TC significantly increased the levels of ROS, while ARV MO displayed a significant reduction in ROS in HepG2 cells. * Represents a significant difference between the control and ARV (***, *p* < 0.0001). α Represents a significant difference between ARV and ARV MO (ααα, *p* < 0.0001).

The effects of ARVs (TDF, FTC, and 3TC: individually) and the protective effects of MO on ROS generation were assessed using the H2DCF-DA assay. All ARV drugs significantly induced the generation of ROS in HepG2 cells, TDF: (*p* = 0.0009), FTC (*p* = 0.0001), 3TC (*p* = 0.0005), while the addition of MO leaf extracts significantly reduced ROS: TDF (*p* = 0.0006), FTC (*p* = 0.0004), 3TC (*p* = 0.0005) (Figure 1).

#### 2.1.2. Cellular Membrane Integrity

**Figure 2:**
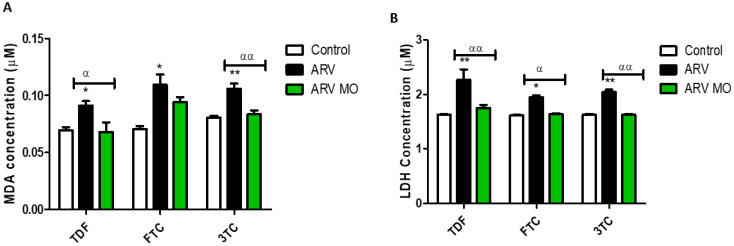
Assessment of cellular membrane integrity post-ARV and post-ARV MO treatment. (**A**) TDF, FTC, and 3TC significantly increased the levels of extracellular MDA, while ARV MO displayed a significant reduction in MDA in HepG2 cells. (**B**) ARVs significantly increased extracellular LDH, whereas ARV MO caused a significant reduction in LDH. * Represents a significant difference between the control and ARV (*, *p* < 0.05 and **, *p* < 0.001). α Represents a significant difference between ARV and ARV MO (α, *p* < 0.05 and αα, *p* < 0.001).

Chronic accumulation of ROS leads to oxidative stress and cell membrane damage; cells form MDA, a biomarker and a by-product of lipid peroxidation, and release LDH. ARVs caused a significant increase in extracellular MDA concentrations: TDF (*p* = 0.0312), FTC (*p* = 0.0137), and 3TC (*p* = 0.0023), while ARV MO reduced MDA levels: TDF (*p* = 0.02171), FTC (*p* = 0.1104), 3TC (*p* = 0.0078) (Figure 2A). In addition, ARVs significantly increased extracellular LDH levels: TDF (*p* = 0.0081), FTC (*p* = 0.0341), and 3TC (*p* = 0.0094), while ARV MO reduced LDH leakage: TDF (*p* = 0.0072), FTC (*p* = 0.0245), 3TC (*p* = 0.0061) (Figure 2B).

### 2.2. Mitochondrial Integrity

**Figure 3:**
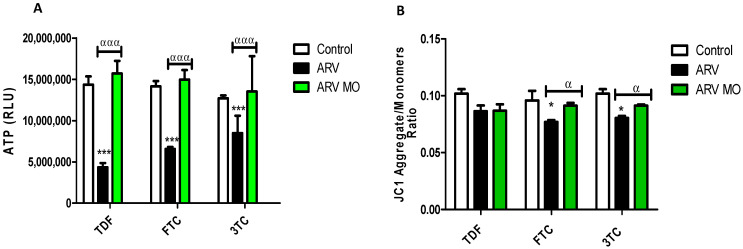
Assessment of mitochondrial integrity post-ARV and post-ARV MO treatment. (**A**) TDF, FTC, and 3TC significantly decreased the levels of intracellular ATP, whereas ARV MO displayed a significant increase in ATP in HepG2 cells. (**B**) ARVs significantly decreased the ΔΨm. In contrast, ARV MO significantly increased ΔΨm in HepG2 cells. * Represents a significant difference between the control and ARV (*, *p* < 0.05 and ***, *p* < 0.0001). α Represents a significant difference between ARV and ARV MO (α, *p* < 0.05 and ααα, *p* < 0.0001).

The intracellular concentration of ATP and mitochondrial membrane potential (ΔΨm) was measured using an ATP luminescence assay and a membrane permeable potentiometric dye, JC-1, respectively, in ARV and ARV MO treated cells. ARVs resulted in a significant decrease in intracellular ATP concentration: TDF (*p* = 0, 0002), FTC (*p* = 0.0007), 3TC (*p* = 0.0002) while ARV MO significantly increased ATP levels: TDF (*p* = 0.0001), FTC (*p* = 0.0001), 3TC (*p* = 0.0006) (Figure 3A). ARVs caused a significant decrease in ΔΨm: TDF (*p* = 0.0841), FTC (*p* = 0.0168), and 3TC (*p* = 0.0229); however, ARV MO increased ΔΨm: TDF (*p* = 0.0631), FTC (*p* = 0.0291), 3TC (*p* = 0.0211) (Figure 3B).

### 2.3. Intracellular GSH Expression

**Figure 4:**
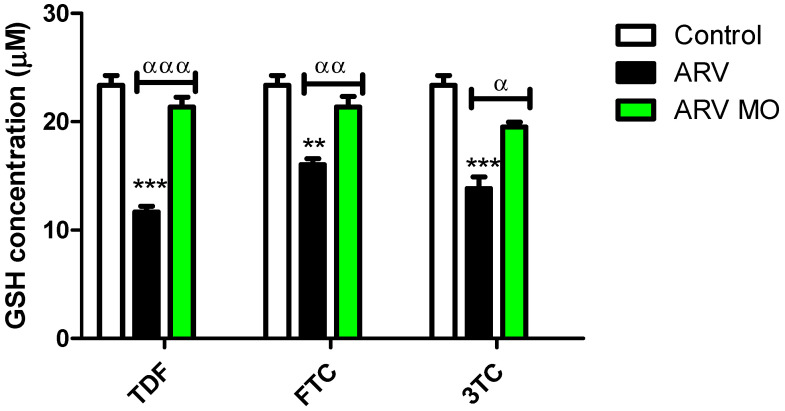
Assessment of GSH concentration post-ARV and post-ARV MO treatment. TDF, FTC, and 3TC significantly decreased the levels of intracellular GSH, while ARV MO displayed a significant increase in GSH in HepG2 cells. * Represents a significant difference between the control and ARV (**, *p* < 0.001 and ***, *p* < 0.0001). α Represents a significant difference between ARV and ARV MO (α, *p* < 0.05, αα, *p* < 0.001 and ααα, *p* < 0.0001).

GSH is the initial defense against oxidative stress in cells. This molecule scavenges ROS, dampening oxidative damage to macromolecules [23]. The levels of GSH were measured after treatment with ARVs and ARV MO. ARVs resulted in a significant decrease in intracellular GSH concentration: TDF (*p* = 0.0003), FTC (*p* = 0.0013), and 3TC (*p* = 0.0013). On the other hand, ARV MO significantly increased GSH: TDF (*p* = 0.0005), FTC (*p* = 0.0036), and 3TC (*p* = 0.0138) (Figure 4).

### 2.4. NRF2 Signalling Pathway and Related Antioxidants

#### 2.4.1. NRF2 Expression

**Figure 5:**
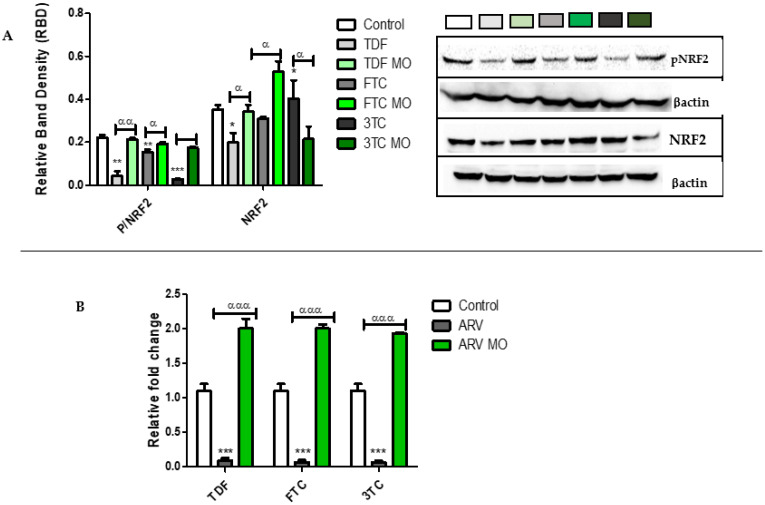
Assessment of pNRF2 and NRF2 expression post-ARV and post-ARV MO treatment. (**A**) TDF, FTC, and 3TC significantly decreased the levels of pNRF2, TDF and 3TC significantly decreased the expression of NRF2, while 3TC caused a slight increase in NRF2 expression. MO increased pNRF2 and NRF2 in ARVs treatments with a slight decrease at 3TC. (**B**) The *NRF2* mRNA was significantly decreased by TDF, FTC, and 3TC while significantly increased by ARV MO. * Represents a significant difference between the control and ARV (*, *p* < 0.05, **, *p* < 0.001 and ***, *p* < 0.0001). α Represents a significant difference between ARV and ARV MO (α, *p* < 0.05, αα, *p* < 0.001 and ααα, *p* < 0.0001).

NRF2 is stimulated and phosphorylated to its active form (pNRF2) during oxidative stress. pNRF2 translocates to the nucleus and binds to the ARE, thus, allowing the transcription of several antioxidant genes. The protein expression (Figure 5A) of NRF2 and pNRF2 and mRNA expression (Figure 5B) of *NRF2* was determined in untreated and treated (ARV and ARV MO) cells. ARVs significantly downregulated pNRF2: TDF (*p* = 0.0064), FTC (*p* = 0.0072), 3TC (*p* = 0.0002). Further, ARVs decreased protein expression of NRF2: TDF (*p* = 0.0306) and FTC (*p* = 0.05015), with a slight increase in 3TC (*p* = 0.0293) and decreased *NRF2* mRNA: TDF (*p* = 0.0009), FTC (*p* = 0.0001), 3TC (*p* = 0.0005). Interestingly, ARV MO significantly increased pNRF2 expression: TDF (*p* = 0.0012), FTC (*p* = 0.0330), and 3TC (*p* = 0.0375). In addition, ARV+MO significantly increased NRF2 protein: TDF (*p* = 0.0072) and FTC (*p* = 0.0001), with a decrease at 3TC (*p* = 0.0508), and significantly increased *NRF2* mRNA: TDF (*p* = 0.0002) and FTC (*p* = 0.0001), 3TC (*p* = 0.0006).

#### 2.4.2. Cytoprotective Antioxidant Enzymes

**Figure 6:**
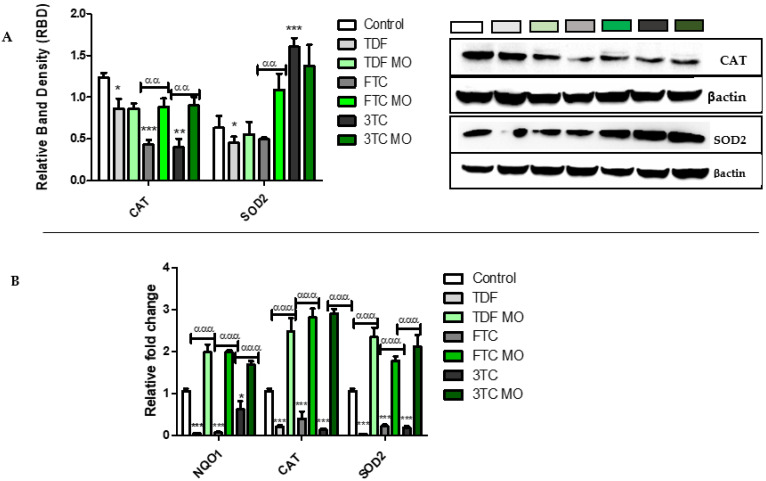
Assessment of antioxidants CAT, SOD2, and NQO1 expression post-ARV and post-ARV MO treatment. (**A**) CAT was significantly decreased by TDF, FTC, and 3TC while significantly increased by MO. SOD2 was significantly decreased by TDF and FTC and increased by 3TC. MO significantly increased SOD2 expression at TDF and FTC; however, there was a slight decrease in MO and 3TC treated cells (**B**) The *CAT, SOD2,* and *NQO1* mRNA was significantly decreased by TDF, FTC, and 3TC while increased by the addition of MO. * Represents a significant difference between the control and ARV (*, *p* < 0.05, **, *p* < 0.001 and ***, *p* < 0.0001). α Represents a significant difference between ARV and ARV MO (αα, *p* < 0.001 and ααα, *p* < 0.0001).

The translocation of pNRF2 to the nucleus allows its binding to ARE and the transcription of antioxidants such as CAT, SOD2, and NQO1. The protein expression of CAT and SOD2 (Figure 6A) and mRNA expression of *CAT*, *SOD2*, and *NQO1* was examined (Figure 6B) post treatments. ARVs significantly decreased CAT protein: TDF (*p* = 0.0389), FTC (*p* = 0.0004), 3TC (*p* = 0.0011) as well as *CAT* mRNA: TDF (*p* = 0.0007), FTC (*p* = 0.0004), 3TC (*p* = 0.0375). Further, ARVs decreased SOD2 protein: TDF (*p* = 0.0414), FTC (*p* = 0.0515), with an increase at 3TC (*p* = 0.0008) and decreased *SOD2* mRNA: TDF (*p* = 0.0001), FTC (*p* = 0.0002), 3TC (*p* = 0.0004), lastly, ARVs significantly decreased *NQO1* mRNA: TDF (*p* = 0.0002), FTC (*p* = 0.0001), 3TC (*p* = 0.0002), Captivatingly, ARV MO increased CAT protein: TDF (*p* = 0.0632), FTC (*p* = 0.0060), 3TC (*p* = 0.0036) as well as *CAT* mRNA: TDF (*p* = 0.0004), FTC (*p* = 0.0004) 3TC (*p* = 0.0001). ARV MO further increased SOD2 protein: TDF (*p* = 0.0563), and FTC (*p* = 0.0038), while showing a slight decrease at 3TC (*p* = 0.0727) and *SOD2* mRNA: TDF (*p* = 0.0009), FTC (*p* = 0.0007), 3TC (*p* = 0.0002). Lastly, ARV MO significantly increased *NQO1* mRNA expression: TDF (*p* = 0.0002), FTC (*p* = 0.0008), and 3TC (*p* = 0.0003).

#### 2.4.3. Mitochondrial Protective Enzymes

**Figure 7:**
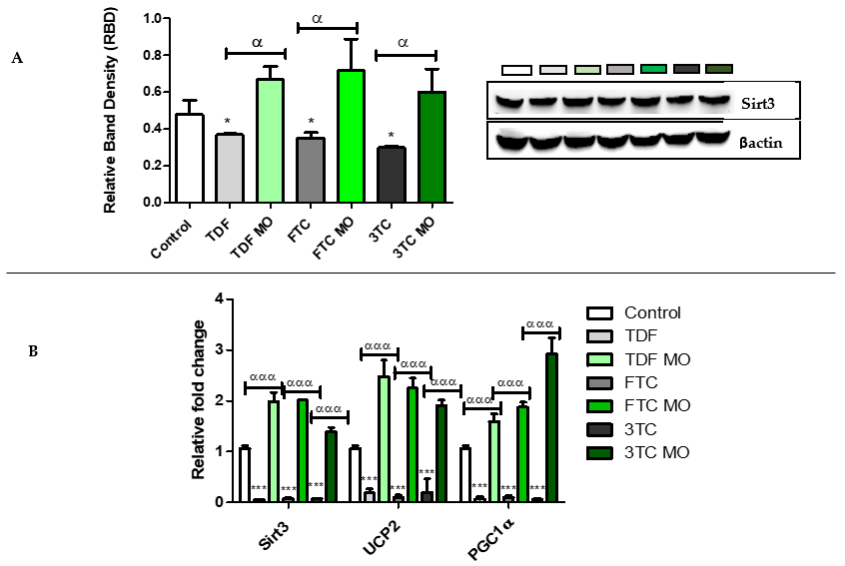
Assessment of mitochondrial protective enzymes expression post-ARV and post-ARV MO (**A**) Sirt3 expression was significantly decreased by TDF, FTC, and 3TC and significantly increased by ARV MO. (**B**) The mRNA of *Sirt3, UCP2,* and *PGC1α* was significantly decreased by ARVs and increased by ARV MO. * Represents a significant difference between the control and ARV (**, *p* < 0.001 and ***, *p* < 0.0001). α Represents a significant difference between ARV and ARV MO (αα, *p* < 0.001 and ααα, *p* < 0.0001).

The mitochondrial response to oxidative stress was evaluated by quantifying protein (Figure 7A) and mRNA (Figure 7B) expression of Sirt3. Further mRNA expression of *PGC1α* and *UCP2*, the regulators of mitochondria biogenesis, was assessed. ARVs decreased Sirt3 protein significantly: TDF (*p* = 0.0353), FTC (*p* = 0.0279), 3TC (*p* = 0.0197) as well as *Sirt3* mRNA: TDF (*p* = 0.0004), FTC (*p* = 0.0004), 3TC (*p* = 0.0391). Similarly, ARVs significantly decreased *UCP2* mRNA: TDF (*p* = 0.0003), FTC (*p* = 0.0003), and 3TC (*p* = 0.0002). Lastly, ARVs significantly decreased *PGC1α* mRNA: TDF (*p* = 0.0006), FTC (*p* = 0.0003), and 3TC (*p* = 0.0007). In contrast, ARV MO increased Sirt3 significantly at the protein level: TDF (*p* = 0.0073), FTC (*p* = 0.0045), 3TC (*p* = 0.0062), and mRNA level: TDF (*p* = 0.0005), FTC (*p* = 0.0004), 3TC (*p* = 0.0012). The same trend was observed with *UCP2* in ARV MO-treated cells: TDF (*p* = 0.0003), FTC (*p* = 0.0006), and 3TC (*p* = 0.0005). Finally, ARV MO significantly increased *PGC1α* mRNA: TDF (*p* = 0.0009), FTC (*p* = 0.0001), and 3TC (*p* = 0.0005).

## 3. Discussion

Regardless of its high efficacy in suppressing HIV viral replication, HAART cannot completely eliminate the virus [61]; therefore, HIV-infected patients need to be on lifetime treatment to achieve low (less than 50 copies/mL) plasma HIV RNA levels [62,63]. Although the new generation of ARV drugs is considered much safer, they are not devoid of adverse effects [64,65]. Hence, it is important to explore novel, economical, and safe supplements/compounds to help attenuate chronic ARV-induced toxicity and adverse effects.

Previous studies showed that MO leaf extracts could prevent, protect and reduce oxidative stress in both in vitro and in vivo models. In this study, we determined the toxicity of ARVs and the potential of MO to ameliorate this ARV-induced toxicity by focusing on oxidative stress, particularly the NRF2 signaling response.

Our data show that all ARV treatments induced oxidative stress in HepG2 cells. TDF, FTC, and 3TC significantly increased the concentration of ROS in liver cells over 96 h, with a concomitant decrease in GSH levels. This implies that increased levels of ROS exceeded the metabolic capabilities of the primary antioxidant GSH to neutralize toxic metabolites and maintain glutathione in the reduced form. These results agree with previous studies on ARV drugs, which showed that TDF, FTC, and 3TC induced oxidative stress [31,66,67]. In rats, TDF was shown to deplete GSH levels [31,68].

There are many benefits of using medicinal plants as supplements to ameliorate the adverse effects of chronic drug therapy. These medicinal plant extracts have the capacity, amongst other beneficial properties, to maintain cellular and tissue redox balance. While all ARV drugs significantly increased ROS generation in HepG2 cells, MO leaf extracts could reduce ROS production; also, MO allowed the build-up of GSH, which was severely depleted by the ARVs. This protective effect is due to MO antioxidant activity, as the extracts contain bioactive polyphenols (catechin, quercetin, and kaempferol) known to combat ROS and prevent oxidative damage [69]. Yetuk, Pandir [70], Chen, Zhou [71] revealed that catechin polyphenols act as antioxidants by eliminating free radicals and chelating surplus free radicals. A study on 1,1-diphenyl-2-picrylhydrazyl (DPPH)-2,2-diphenyl-1-picrylhydrazyl (DPPH), a free radical that has hydrogen acceptor capability to antioxidants showed that MO extracts exhibited high DPPH-scavenging activity [41]. Furthermore, MO contains quercetin (hydroxyphenyl groups) and other flavonoids, which display potent antioxidant effects by inhibiting the production of ROS and reactive nitrogen species [72,73].

Prolonged and excessive generation of ROS can damage macromolecules. The primary phase of ROS-mediated cellular damage is the peroxidation of cell membrane lipids and leakage of the cytoplasmic enzyme LDH [74]. TDF, FTC, and 3TC increased both the formation of MDA (a by-product of lipid peroxidation) and LDH leakage in HepG2 cells. Similar trends were reported in previous in vivo studies [31,75]. MO counteracted oxidative stress and prevented lipid peroxidation and cellular membrane damage. Another study also showed that MO leaf extracts reduced ROS levels and extracellular MDA concentrations significantly [76].

Further, ROS accumulation can have a negative impact on mitochondrial proteins and DNA by altering the electrochemical gradient across the mitochondrial membrane resulting in mitochondrial dysfunction [77,78]. ARV drugs decreased the ΔΨm in HepG2 cells; in addition, ATP levels were significantly decreased. Mitochondria need an electrochemical gradient for ATP synthesis. Thus, a decrease in ΔΨm severely compromises ATP production, leads to a change in permeability, and promotes mitochondrial swelling. Studies reported that TDF, FTC, and 3TC induced mitochondrial toxicity by decreasing ΔΨm, inhibiting OXPHOS complex I and complex IV enzymes, decreasing oxygen consumption, and increasing the production of mitochondrial ROS as well as ATP synthesis impairment [32,33,79,80,81,82,83].

In contrast to ARV drugs, MO displayed the potential to mitigate mitochondrial impairment, as confirmed by the improved ΔΨm and ATP levels. MO leaf extracts were shown to have neuroprotective effects via antioxidative and mitochondrial regulation in human neuroblastoma cells [84]. In a previous study, MO minimized impaired mitochondria by improving the mitochondrial NADH dehydrogenase and ATPase enzyme activity; the study also showed that MO leaf extract attenuates high glucose-induced metabolism by modulating the mitochondrial respiratory chain in HepG2 cells [48]. MO contains vitamins C (ascorbic acid) and E (α-tocopherol), which can improve oxidative phosphorylation and protect the mitochondrial membrane from peroxidation, respectively [73].

Cells respond to oxidative stress by activating NRF2, a master regulator of the antioxidant response [25]; it resides in the cytoplasm by constitutive degradation through CUL3 ubiquitin ligase complexes using the protein KEAP1 as a substrate adaptor. Activation of NRF2 causes it to translocate into the nucleus to initiate transcription of the ARE. NRF2 activates several genes which encode antioxidant proteins [85]; these NRF2-target genes include NAD(P)H NQO1, SOD2, CAT [86,87] and Sirt3 [88]. TDF, FTC, and 3TC decreased pNRF2 as well as NRF2 mRNA and protein. ARVs decreased *NQO1* expression, a flavin co-factor, and a flavoprotein that functions as a superoxide reductase and plays a role in the direct scavenging of superoxide anions [89]. TDF, FTC, and 3TC significantly reduced the mRNA and protein expression of *CAT*. CAT mitigates oxidative stress by converting hydrogen peroxide to water molecules. This study reveals that ARVs negatively regulate the NRF2 pathway. Similar to our study, Similar to our study, Sibiya, Ghazi [90] showed a significant decrease in NRF2 and pNRF2 protein expression in HepG2 cells treated with ARV drugs (TDF and 3TC). Singh, Kotla [91] showed that the HAART (containing TDF and FTC) increased monocyte/macrophage sensitivity to ROS in HIV^+^ individuals by suppressing NRF2-ARE activity via p90RSK-mediated ERK5 S496 phosphorylation.

We observed a significant decrease in mitochondrial SOD2, Sirt3, and PGC1α expression by TDF, FTC, and 3TC. PGC1α, a transcriptional co-activator and a potent regulator of cellular metabolism, maintain the balance between the production and neutralization of oxidants by regulating mitochondrial biogenesis and antioxidant gene expression [92]. The downregulation of PGC1α negatively affects Sirt3 expression. Sirt3 controls NAD+-dependent mitochondrial substrate deacetylation and attenuates ROS by deacetylating and activating SOD2 [93,94,95]. The downregulation of PGC1α also has negative feedback on UCP2 transcription. UCP2 protects against oxidative stress by improving the mitochondrial NAD^+^/NADH ratio by suppressing ROS generation, and the NAD^+^ levels directly control Sirt3 activity. The findings on the mitochondrial antioxidant response agree with the downregulation of the NRF2 antioxidant pathway in the consumption of TDF, FTC, and 3TC for a longer period.

Previous in vivo studies reported depletion of SOD1 and SOD2 by TDF in male Wistar rat kidney tissues [31]. ElZohary, Weglicki [96] suggested that the downregulation of NRF2 severely compromised the antioxidant response in HAART (containing TDF and FTC) treated rats. Previous studies also reported a significant decrease in SOD and CAT post-treatment with TDF [68]

**Figure 8:**
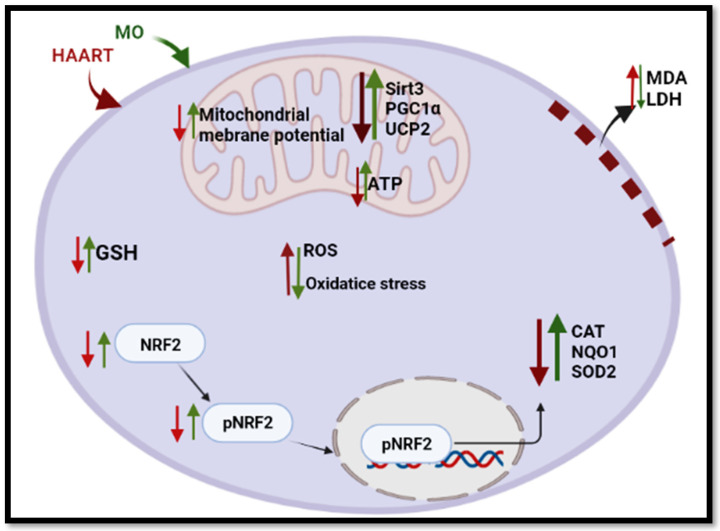
A brief overview of MO’s potential to ameliorate the long-term HAART-induced toxicity in HIV-positive individuals. HAART depolarises the mitochondrial membrane and reduces ATP. HAART increases ROS and reduces GSH and other antioxidants, therefore promoting oxidative stress. The addition of MO attenuates ARV toxicity by increasing the polarisation of the mitochondria and ATP synthesis. MO scavenges ROS and restores GSH levels. MO upregulates the NRF2 antioxidant pathway and further reduces oxidative stress. MO leaf extracts reduce MDA and LDH levels by reducing the peroxidation of lipids. Created in Biorender.com (access date: 14 June 2022).

MO attenuated ARV toxicity by upregulating the expression of NRF2 and its related antioxidants. This effect was previously demonstrated at the transcriptional and translational levels in various cell lines, including HepG2, HK-2, and V79-MZ cells, as well as animal models [97,98,99], C2C12 skeletal muscle cells [100] as well as in rat kidneys [101,102] and rat liver tissues [103,104]. MO contains several bioactive compounds, such as quercetin glucosinolates, isothiocyanate, flavonoids, and phenolic acids, that have been found to activate the NRF2-ARE [36,99,105]. For example, sulforaphane is a glucosinolate glucoraphanin-derived isothiocyanate. This compound induces many cytoprotective proteins, including antioxidant enzymes such as heme oxygenase-1 (HO1), NQO1, CAT, SOD, glutathione transferase, gamma-glutamylcysteine ligase, and glutathione reductase, through the NRF2-antioxidant response mechanism [106,107]. Taken together, this study strongly demonstrated ARVs induce mitochondrial impairment and oxidative stress and are toxic to the liver. The study also showed that MO leaf extract potentially ameliorates ARV-induced oxidative stress by (i) allowing the accumulation of GSH and (ii) upregulating the NRF2 signaling pathway (Figure 8). The results of this research have implications for the successful attenuation of ARV-induced toxicity by using MO. Therefore, to improve the metabolism of people on ART, there is a need to incorporate an adjuvant therapy with the current antiretroviral treatment in the form of an extract or phytochemical(s) derived from MO leaves.

## 4. Materials and Methods

ARV drugs (TDF, FTC, and 3TC) were purchased from Toronto Research Chemicals Inc. (North York, ON, Canada). MO leaves were obtained from Durban (KwaZulu-Natal, South Africa and authenticated by the University herbarium (Batch no. CT/1/2012, Genus no. 3128)). HepG2 cells were purchased from American Type Culture Collection (ATCC; Johannesburg, Gauteng, South Africa). Cell culture reagents were purchased from Whitehead Scientific (Johannesburg, Gauteng, South Africa). Western blot reagents were purchased from Bio-Rad (Johannesburg, Gauteng, South Africa). All other reagents were purchased from Merck (Johannesburg, Gauteng, South Africa) unless otherwise stated. All results were verified by performing two independent experiments in triplicate.

### 4.1. Moringa oleifera Lam Leaf Extracts Preparation

MO leaves were collected, air-dried, and crushed in a pestle and mortar. A volume of 100 mL deionized water was added, and the resultant extract was boiled with continuous stirring (20 min (min)), transferred to 50 mL conical tubes, and centrifuged [720 g, 10 min, room temperature (RT)]. The aqueous upper layer (MO extract) was then removed, filter sterilized [0.22-µm filter (Millipore, GVWP04700, Merck (Johannesburg, Gauteng, South Africa)], lyophilized, and stored at 4 °C. For subsequent assays, MO extracts stock solution was prepared by dissolving in 0.1 M phosphate-buffered saline (PBS). Gas chromatography-mass spectrometry was obtained from a recent analysis performed within the lab [108]. High-Performance Liquid Chromatography (HPLC) analysis of aqueous MO leaf extract was obtained from [109,110,111,112,113].

### 4.2. Drug Solutions and Treatment Preparation

Mean steady-state peak plasma concentration (C_max_) is the most physiologically relevant concentration for the ARVs. C_max_ was used to treat cells because it represents naturally occurring concentrations of the drugs following their consumption [114]. C_max_ concentrations for the ARV drugs were as follows: FTC C_max_ = 1.8 µg/mL, TDF C_max_ = 0.3 µg/mL, and 3TC C_max_ = 1.5 µg/mL [115]. First, ARV drugs were dissolved in 5 mL sterile dH_2_O and thereafter diluted in Eagle’s Minimum Essentials Medium (EMEM) to obtain the desired C_max_. The total ARV drugs incubation time 96 h (h).

### 4.3. Cell Culture and Treatments

HepG2 cells were cultured in 25 cm^3^ sterile cell culture flasks as a monolayer containing complete culture media (CCM), EMEM, 10% fetal calf serum, 1% penicillin-streptomycin fungizone, and 1% L-glutamine), until 60–70% confluent. Cells were treated with ARVs for 96 h, and MO was added to the cells post-ARV treatment for 24 h. In all treatments, CCM was replenished every 48 h. MO extract concentration for HepG2 cells was optimized through a cell viability test by assessing a set of MO concentrations extrapolated from [108]. The MO concentration (750 µg/mL) was optimized for the antioxidant response in HepG2 cells. Concentration-dependent effects (0, 50, 100, 250, 500, 750, 1000, 1250 µg/mL) were all tested at 6, 24, 48 and 72 h independently. The optimum concentration (750 µg/mL) yielded above 100% cell viability and was used for further experimentation.

### 4.4. Oxidative Stress Assessment

#### 4.4.1. 2′,7′-Dichlorodihydrofluorescein-diacetate (H_2_DCF-DA) Assay

Intracellular ROS was quantified using the fluorometric H_2_DCF-DA assay [116]. Control and treated cells (50,000 cells per treatment) were incubated in 500 μL of 5 μmol/L H_2_DCF-DA stain (30 min, 37 °C). The cells were centrifuged (400× *g*, 10 min, 24 °C) to remove the stain, and cells were washed twice with 0.1 mol/L phosphate buffer saline (PBS). Cells were re-suspended in 400 μL of 0.1 M PBS and seeded in triplicate (100 μL/well) in a 96-well opaque microtiter plate. A blank consisting of only 0.1 M l PBS was plated in triplicate as well. Fluorescence was measured with the Modulus™ microplate luminometer (Turner Biosystems, Sunnyvale, CA, USA) using a blue filter with an excitation wavelength (λex) of 503 nm and emission wavelength (λem) of 529 nm. The fluorescence of each sample was calculated by subtracting the average fluorescence of the blank from the fluorescence of each sample.

#### 4.4.2. Thiobarbituric acid Reactive Substances (TBARS) Assay

Lipid peroxidation by-products—malondialdehyde (MDA) was measured using the TBARS assay. This assay was conducted as previously described [117]. Sample absorbance was read using a spectrophotometer, λ = 532/600 nm. The TBARS results are expressed in terms of MDA concentration.

#### 4.4.3. Lactate Dehydrogenase (LDH) Assay

Extracellular levels of LDH were assessed using the LDH Cytotoxicity Detection Kit [118] (11644793001; Roche, Mannheim, Germany). The supernatants (100 µL) from control and treated cells were added into a 96-well microtiter plate in triplicate. The substrate mixture (100 µL) consisting of a catalyst (diaphorase/NAD+) and a dye solution (INT/sodium lactate) was added to the supernatant and incubated at RT for 25 min. Optical density was measured at 500 nm using a microplate reader (Bio-Teck µQuant). Results are presented as mean optical density.

### 4.5. Mitochondrial Integrity

#### 4.5.1. Adenosine Triphosphate (ATP) Assay

CellTitre Glo™, MAK1901KT (Promega, Madison, CA, USA) assay was used to assess intracellular ATP levels [119]. Briefly, 50 μL of cell suspension (20,000 cells/well in 0.1 M PBS) was seeded into a white, opaque 96-well plate in triplicate. CellTitre Glo™ reagent (20 μL) was added into each well, followed by incubation of the plate in the dark (30 min) at RT. Luminescence, which is linearly related to the levels of intracellular ATP, was detected using a Modulus™ microplate luminometer (Turner Biosystems, Sunnyvale, CA, USA). Results are presented as relative light units (RLU).

#### 4.5.2. Mitochondrial Membrane Potential

The mitochondrial membrane potential (Δψm) was measured using the JC-1 stain. Control and treated cells (50,000 cells) were incubated in 200 μL of 5 μg/mL JC-1 stain (BD Biosciences, San Jose, NJ, USA) (20 min, 37 °C). The stain was removed via centrifugation (400× *g*, 10 min, 24 °C), and the cells were washed twice with JC-1 staining buffer. Cells were re-suspended in 400 μL of JC-1 staining buffer and seeded in an opaque 96-well plate in triplicate (100 μL/well). A blank, which consisted of only JC-1 staining buffer, was plated in triplicate (100 μL/well). Fluorescence was quantified on a Modulus™ microplate reader (Turner Biosystems, Sunnyvale, CA, USA). JC-1 monomers were measured with a blue filter (λex = 488 nm, λem = 529 nm), and JC-1 aggregates were measured with a green filter (λex = 524 nm, λem = 594 nm). The Δψm of the HepG2 cells was expressed as the fluorescence intensity ratio of JC-1 aggregates and JC-1 monomers [120].

### 4.6. Antioxidants Assays

#### 4.6.1. Glutathione Assay

The GSH-Glo™ assay (V6912, Promega, Madison, WI, USA) was used to quantify total and reduced glutathione. Cells were transferred to a white microtiter plate (50 μL of 20,000 cells/well: 3 replicates). GSH standards (0–5 μM) were prepared from a stock solution diluted in deionized water (5 mM). The 2X GSH-Glo™ Reagents were prepared according to the manufacturer’s instructions, added to the experimental wells (50 μL/well), and incubated (RT, 30 min). Reconstituted Luciferin Detection Reagent (50 μL) was added to each well and incubated (RT, 15 min) before the luminescence was measured (Modulus™ microplate luminometer, Turner Biosystems, Sunnyvale, CA, USA). A standard curve was constructed using GSH standards, and lastly, GSH concentrations (μM) in both control and treated cells were extrapolated [121].

#### 4.6.2. Western Blots

Protein expression of NRF2, pNRF2, CAT, SOD2, Sirt3, and UCP2 was quantified using western blots [122]. Briefly, Cytobuster™ Reagent (250 μL) (Novagen, San Diego, CA, USA, catalog no. 71009) supplemented with protease and phosphatase inhibitors (Roche; 05892791001 and 04906837001, respectively) was used for protein extraction. Protein samples were quantified using the bicinchoninic acid (BCA) assay and standardized to 1 mg/mL in a cytobuster. Standardized protein samples were heated (100 °C) for 5 min in Laemmli sample buffer [dH2O, 0.5 M Tris-HCl (pH 6.8), glycerol, 10% SDS, β-mercaptoethanol, 1% bromophenol blue]. Prepared protein samples were electrophoresed in sodium-dodecyl sulphate polyacrylamide gels (SDS-PAGE) (4% stacking gel, 10% resolving gel) for 1.5 h at 150 V (Bio-Rad Mini PROTEAN Tetra-Cell System). Separated proteins were electro-transferred from the gel onto nitrocellulose membranes using the Trans-Blot Turbo Transfer System (standard mixed protein program, Bio-Rad). The membranes were blocked with 5% bovine serum albumin (BSA) in Tris Buffer Saline with tween 20 (TTBS- NaCl, KCl, Tris, dH2O, 0.5% tween 20, pH 7.5) or 5% Non-Fat Dry Milk (NFDM) in TTBS for phosphorylated and non-phosphorylated antibodies, respectively. Membranes were incubated with primary antibody (1:1000; Table 1) overnight (4 °C). The membranes were washed five times with TTBS (10 min, RT) and probed with a horse-radish peroxidase (HRP)-conjugated secondary antibody [goat anti-rabbit (Cell Signalling Technology, #7074S; 1:5000) 1 h, RT]. The membranes were washed five times again in TTBS (10 min, RT). The Clarity™ Western ECL Substrate Kit (Bio-Rad, #170-5060) was used to detect specific protein bands, and the images were captured using the ChemiDoc™ XRS+ Molecular Imaging System (Bio-Rad). The membranes were then stripped using hydrogen peroxide (5%, 37 °C, 30 min), washed once in TTBS (10 min, RT), and probed with the housekeeping protein, anti-β-actin (Sigma-Aldrich, A3854; 1:5000; 30 min, RT) to normalize protein expression. Analysis was performed using the Bio-Rad Image Lab Software version 5.1, and the results were represented as relative band density (RBD).

#### 4.6.3. qPCR

RNA was isolated from control and treated HepG2 cells using an in-house protocol [127]. Total RNA was quantified (Nanodrop 2000, Thermo Fischer, Johannesburg, Gauteng, SA) and standardized to (1000 ng/mL). From the standardized RNA samples, complementary DNA (cDNA) was generated using a commercially available kit (iScript™ cDNA Synthesis kit, Bio-Rad; catalog no 107-8890). A 20 μL reaction volume containing 4 μL 5X iScript™ reaction mix,1 μL iScript reverse transcriptase 1 μL RNA template, and 14 µL nuclease-free water was prepared. Thermocycler conditions were 25 °C for 5 min, 42 °C for 30 min, 85 °C for 5 min, and a final hold at 4 °C.

The mRNA levels of genes (Table 2) related to the antioxidant response (*NRF2*, *SOD2*, *CAT*, *NQO1*, *Sirt3*, *PGC1a*, and *UCP2*) were assessed using the Sso Advanced™ Universal SYBR^®^ Green SuperMix (Bio-Rad, catalog no. 172-5271) on the CFX Touch™ Real-Time PCR Detection System (Bio-Rad, Hercules, CA, USA). Each measurement was taken in triplicate and normalized against GAPDH, which was amplified under the same conditions. Data were analyzed using the Bio-Rad CFX Manager™ Software version 3.1. The comparative threshold cycle (Ct) method was used to determine relative changes in expression [128].

### 4.7. Statistical Significance

For statistical analyses, GraphPad Prism version 5.0 (GraphPad Software Inc., San Diego, CA, USA) was used. The unpaired *t*-test with Welch’s Correction test was used to determine the statistical differences among the groups. Data were represented as the mean ± standard deviation (*n* = 3). A statistically significant *p*-value was less than 0.05. * Represents Control vs. ARV, α Represents ARV vs. ARV MO. All results were verified by performing two independent experiments in triplicate.

## 5. Conclusions

This study reveals that the long-term use of HAART may pose a certain degree of toxicity, mainly through oxidative stress and mitochondrial toxicity hence altering metabolic and physiological conditions, putting people consuming HAART at risk of liver injury. However, the use of MO leaf extracts targeting the NRF2 antioxidant signaling mechanism attenuates HAART-induced toxicity. More extensive research in animal models as well as the synergistic relationship between MO and HAART, may be considered for future study. The limitation of in vitro models is that they usually consist of a single monolayer of cells (HepG2 cells) and have lower metabolic profiles, hence limiting interpretations of interactions between the various cell types found in a multicellular organism.

## Figures and Tables

**Table 1:** Primary antibodies used for protein expression.

Antibody	Catalog Number	Reference
pNRF2	Abcam, (ab76026) Rabbit mAb	[123]
NRF2	Cell signaling, Rabbit mAb #12721	[124,125]
SOD2	Cell signaling, Rabbit mAb #13194	[125]
CAT	Cell signaling, Rabbit mAb #14097	[121]
Sirt3	Cell signaling, Rabbit mAb #5490	[126]

**Table 2:** Primer sequences and annealing temperatures of the genes of interest.

Gene	Primer Sequences	Annealing Temperature (°C)
*NRF2*	Sense 5′CACATCCAGTCAGAAACCAGTGG3′ Antisense 5′GGAATGTCTGCGCCAAAAGCTG3′	60
*SOD2*	Sense 5′CTGGACAAACCTCAGCCCTAAC3′ Antisense 5′AACCTGAGCCTTGGACACCAAC3′	57
*Sirt3*	Sense 5′CCCTGGAAACTACAAGCCCAAC3′ Antisense 5′GCAGAGGCAAAGGTTCCATGAG3′	58
*PGC1α*	Sense 5′CCAAAGGATGCGCTCTCGTTCA3′ Antisense 5′CGGTGTCTGTAGTGGCTTGACT3′	62
*NQO1*	Sense 5′CCTGCCATTCTGAAAGGCTGGT3′ Antisense 5′GTGGTGATGGAAAGCACTGCCT3′	58
*CAT*	Sense 5′GTGCGGAGATTCAACACTGCCA3′ Antisense 5′CGGCAATGTTCTCACACAGACG3′	60
*UCP2*	Sense 5′TGGTCGGAGATACCAAAGCACC3′ Antisense 5′GCTCAGCACAGTTGACAATGGC3′	59
*GAPDH*	Sense 5′CACCATTGGCAATGAGCGGTTC3′ Antisense 5′AGGTCTTTGCGGATGTCCACGT3′	Variable

## Data Availability

Not applicable.
